# Supplementary material for: Precise engineering of quantum dot array coupling through their barrier widths
Source: Nat Commun. 2017 Oct 5;8:787. doi: 10.1038/s41467-017-00872-2 (PMC5629208; doi:10.1038/s41467-017-00872-2)
Supplement: Supplementary file 2 — Supplementary info [file 41467_2017_872_MOESM2_ESM.pdf]

Supplementary Methods

EBEM Simulations

For the simulations the first step is to parametrize the geometrical structure of the networks that will mimic the potential barriers that scatter the 2DEG. The complexity of the geometries can be simplified since the molecular electrostatic clouds, that are responsible of the potential barriers, are generally very smooth and relatively featureless. We choose the width and length of the straight beads to match the molecular width (d ~ 5 Å) and the network dimensions. After this parametrization, the V_{eff} and m^* parameters are iterated to fit the experimental data. With V_{eff}, the scattering of the surface state with the molecular barriers is defined and both the band bottom ($\bar{\Gamma}$ point in ARPES and onset of bonding state in STS) and the opening of the gaps are quantitatively determined. The effective mass parameter is inversely proportional to the dispersion of the band so the curvature, i.e. the top of the band or antibonding state, is obtained. Please note that changes in m^* do not alter significantly the energetic position of the band bottom of the first state. Thus, by tweaking these two parameters we can play until we get a perfect agreement with the experimental data. Having ARPES as experimental input facilitates enormously the process since V_{eff} is mainly responsible for the energy shift of the band bottom and m^* is varied to match the band dispersion of n = 1. It is worth noting that this simple fitting procedure works exceptionally well. However, both parameters are slightly inter-linked so small changes in the energy onset or the band width can occur.

Ab initio calculations

In the SW molecular network, the structure is stabilized through purely halogen bonding in a trimer configuration[1] (cf. Supplementary Fig. 3). The halogen atom, when covalently bonded to a molecule, induces an area of positive electrostatic potential on the outermost portion of the atom along the bond axis[1-3], thus stabilizing the network by the attraction between positive and negative electrostatic poles on the Br atoms. The structure transforms in the DW network as halogen bonding is combined with Br···O bonds generating the double molecular barrier between nanocavities.

Synthesis and characterization of Br-DNT and Br-DNF

Br-DNT and Br-DNF were synthesized from new synthetic methodologies, to be published elsewhere. They were purified by multiple recrystallizations and physical vapor transport techniques, and characterized by melting points, ^{1}H NMR, high-resolution mass spectra, and elemental analyses.
All NMR spectra were recorded on JEOL ECS400 spectrometers. Chemical shifts are reported in parts per million (ppm, δ scale) from residual protons in the deuterated solvent for ^{1}H NMR (δ 5.93 ppm for 1,1,2,2-tetrachloroethane) at 100 °C. The data were presented in the following format: chemical shift, multiplicity (s = singlet, d = doublet, t = triplet, m = multiplet), coupling constant in Hertz (Hz), signal area integration in natural numbers, and assignment (in italics). Mass spectra were measured on a Bruker compact mass spectrometer. Melting points and elemental analyses were collected on a Mettler Toledo MP70 Melting Point System and on a J-Science Lab JM10 MICRO CORDER, respectively.

- *3,9-Dibromodinaphtho[2,3-b:2',3'-d]furan (Br-DNF)*. Colorless solid. m.p.: > 300°C. ^{1}H NMR (400 MHz, $CDCl_2CDCl_2$, at 100 °C): 7.55 (dd, *J* = 2.0, 8.8 Hz, 2H, Ar*H*), 7.78 (s, 2H, Ar*H*), 7.88 (d, *J* = 8.8 Hz, 2H, Ar*H*), 8.11 (s, 2H, Ar*H*), 8.45 (s, 2H, Ar*H*). ^{13}C NMR could not be recorded due to the poor solubility. HRMS (APCI+): Calcd for $C_{20}H_{11}OBr_2$ [M+H] 424.9177, found, 424.9155. Anal. Calcd for $C_{20}H_{10}Br_2O$: C, 56.38; H, 2.37, found C, 56.35; H, 2.41.

- *3,9-Dibromodinaphtho[2,3-b:2',3'-d]thiophene (Br-DNT)*. Yellow solid. m.p.: > 300 °C. ^{1}H NMR (400 MHz, $CDCl_2CDCl_2$, at 100 °C): δ 7.60 (dd, *J* = 1.6, 8.8 Hz, 2H, Ar*H*), 7.90 (d, *J* = 8.8 Hz, 2H, Ar*H*), 8.06 (s, 2H, Ar*H*), 8.11 (s, 2H, Ar*H*), 8.64 (s, 2H, Ar*H*). ^{13}C NMR could not be recorded due to the poor solubility. HRMS (APCI+): Calcd for $C_{20}H_{11}SBr_2$ [M+H] 440.8848, found, 440.8843. Anal. Calcd for $C_{20}H_{10}Br_2S$: C, 54.33; H, 2.28, found C, 54.35; H, 2.45.

Supplementary Figures

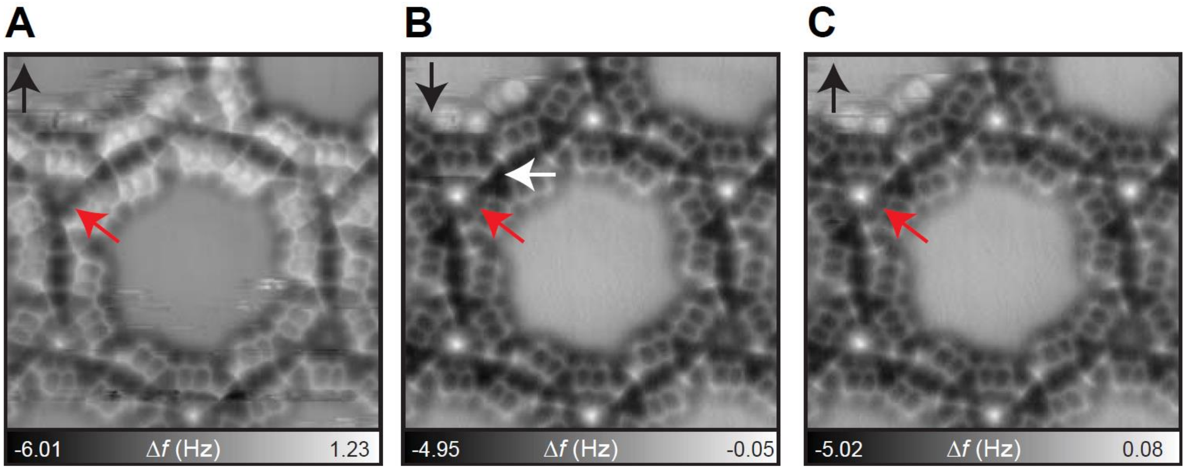

Supplementary Figure 1│Adsorption of CO molecule on the nodal site. (A-C) AFM image series of the DW network when CO is intentionally dosed into the system for the preparation of the CO functionalized tip. Vertical black arrows indicate the slow scan directions. The tip and sample separation in (A) is smaller than those in (B) and (C) by 80 pm. In (A), four CO molecules are found at the nodal sites. By accidental manipulation, the node indicated by the red arrow becomes filled in (B). The horizontal white arrow marks the discontinuous frequency shift detected. Measurement parameters: V = 0 mV, oscillation amplitude A = 60 pm.

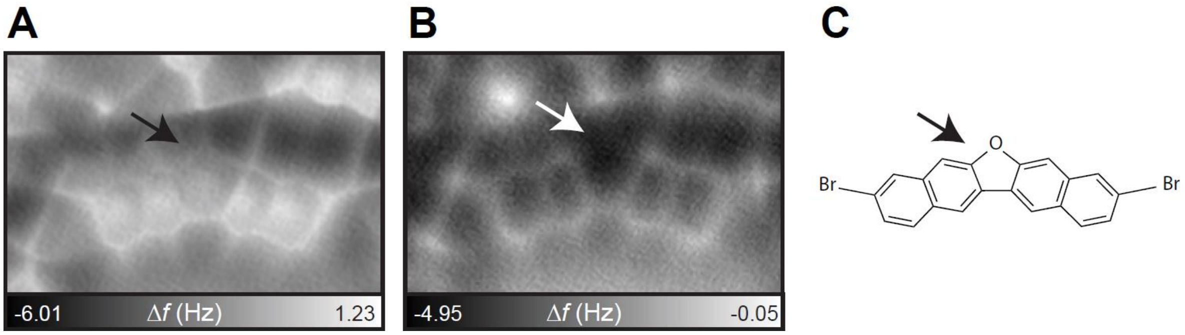

Supplementary Figure 2│Imaging by AFM of the oxygen atom of the furan group. (A-B) Close view AFM image of the furan moiety in Br-DNF, taken in constant height mode at different tip-sample distances. (A) is taken closer than (B) by 80 pm. The O-C bond (C) appears in (A) while it vanishes in (B). The naphthalene moieties are flat on the surface and no steric stress is induced by the adsorption at the furan moiety.

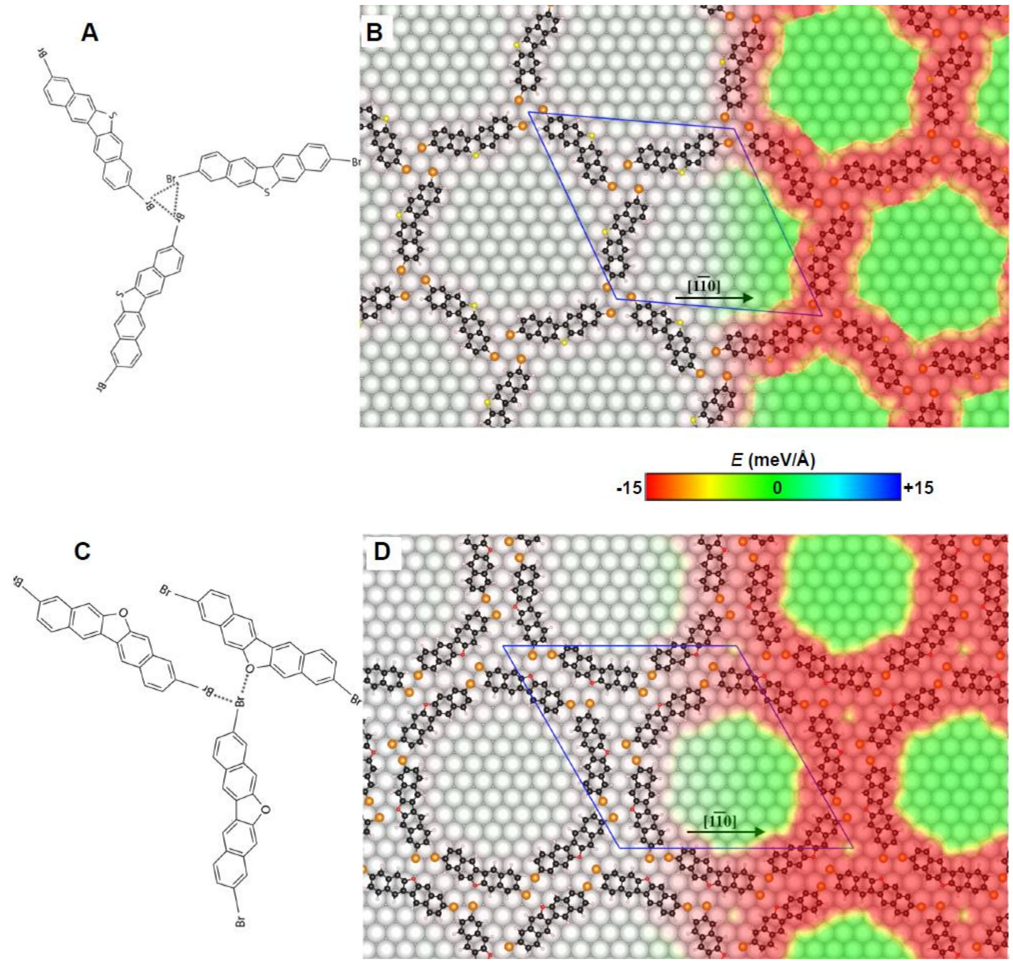

Supplementary Figure 3│Models for SW and DW networks on Ag(111). (A) and (C) Representation of molecular arrangements leading to multiple halogen bonding (dashed lines) between molecular units, and corresponding models predicted by DFT calculations for the arrangement of the SW (B) and DW (D) halogen based networks. Br atoms are shown in brown, O in red, S in yellow and Ag by large gray spheres. The hexagonal lattice of the DW network matches a (12 x 12) surface unit cell. This results in an inter-pore distance of $12a$ = 3.45 nm (a is the Ag-Ag distance) and the molecular layer lattice vector aligns along the $[1\bar{1}0]$ direction. Conversely, the hexagonal lattice of the SW network exhibits a $\begin{pmatrix} 10 & -1 \\ 1 & 11 \end{pmatrix}$ surface unit cell concomitant with atomic positions of the Ag substrate and presenting a small angle of 4.7º with the $[1\bar{1}0]$ direction. The inter-pore distance in this case is $\sqrt{111}a$ = 3.03 nm. The 4 Å difference between the inter-pore distances in the two cases is very close to the width of a molecular block. Therefore, the size of the nanocavities by SW and DW networks are almost identical. The color maps in (B) and (D) illustrate the distribution of the normal component of electric field, E, on a plane 3 Å from the molecular layers (i.e. at the position of the metal surface). The plots share the same color scale, where red corresponds to E < -0.015 V/Å, blue to E > 0.015 V/Å, and green to zero. The local electric field, acting as scatterer for the surface electrons, becomes significant only underneath the molecular units. The effective geometry and width of the barriers is estimated from these plots. The width per molecular unit turns out to be $d \approx 5$ Å, coinciding with the one used in the EBEM models.

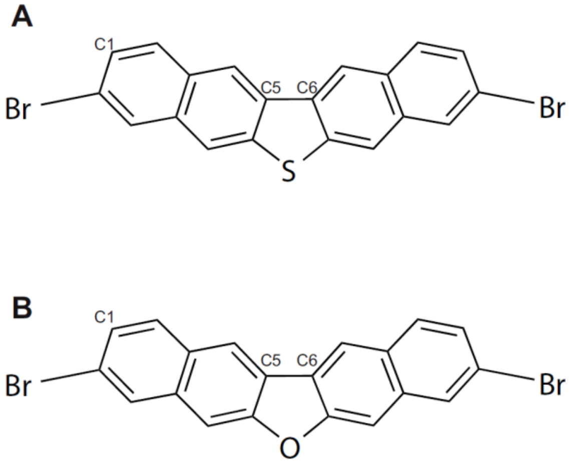

Supplementary Figure 4│DFT calculations of the free Br-DNF and Br-DNT molecules. (A) Schematic drawing of Br-DNF. The C1-C5-C6 angle is 165º with a distance between Br atoms of 1.51 nm. (B) Schematic drawing of Br-DNT. The obtained C1-C5-C6 angle is 159º and the Br-Br separation is 1.52 nm. The lengths of the molecules differ in less than 1%.

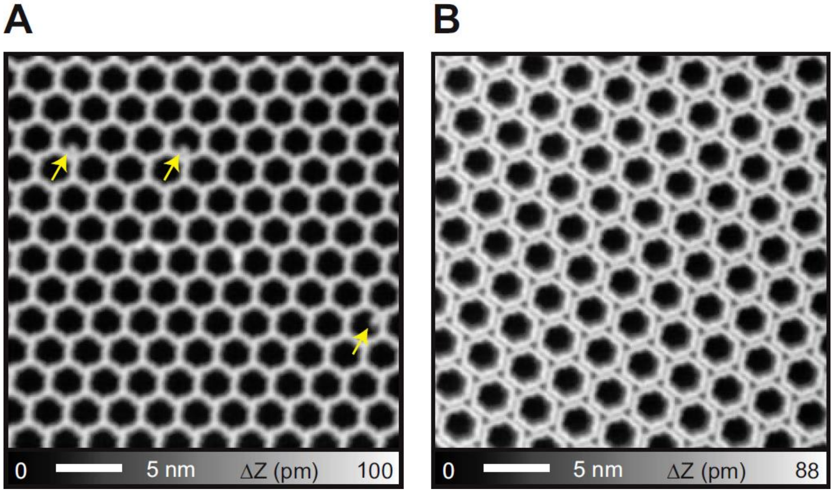

Supplementary Figure 5│Network imperfections after array formation. STM topographic images of SW (A) and DW (B) networks simultaneously recorded with the dI/dV maps shown in Fig. 2B and C of the main text. The yellow arrows mark defects that we assign to excessive Br-DNT at the pore and which modulates its local electronic structure. In (A), only three defective pores out of a total of 132 (2.3%) can be observed. Such amount of imperfections is irrelevant to the ARPES signal.

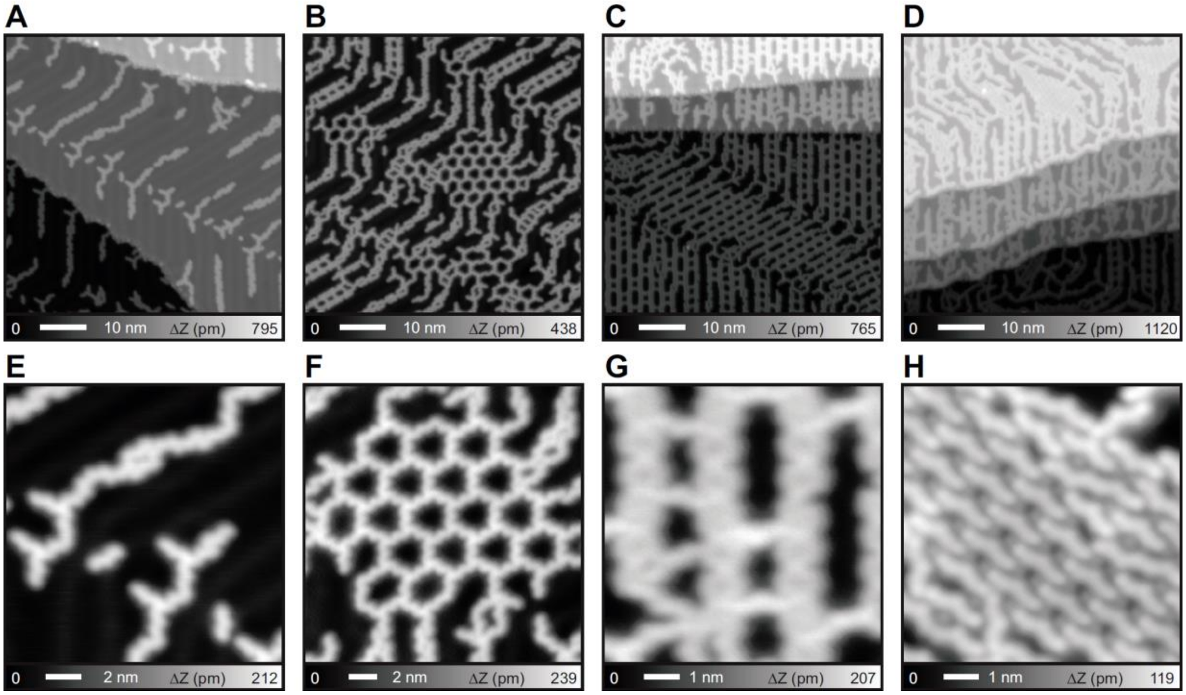

Supplementary Figure 6│Molecular arrangements of Br-DNT deposited on Au(111). (A-D) A series of STM topographic images, with increasing molecular coverage and (E - H) corresponding close-view of them. The surface corrugation prevents the long-range formation of 2D hexagonal molecular networks, facilitating chain structures following the herringbone reconstruction. We stress that if an ideally regular 2D porous network as the one observed in (B) would fully extend over the whole surface, the obtained band structure would be different from the SW and DW cases shown in this work. This is because the pores have different symmetries (three-fold vs six-fold) and exhibits different interpore dimensions, affecting the positions of the high symmetry points in $k_{//}$. Measurement parameters: V_{tip} = -200 mV for all images, I = 10 pA in (A) and (B); I = 2 pA in (C) and (D); I = 5 pA in (E); I = 20 pA in (F); I = 2 pA in (G); I = 20 pA in (H).

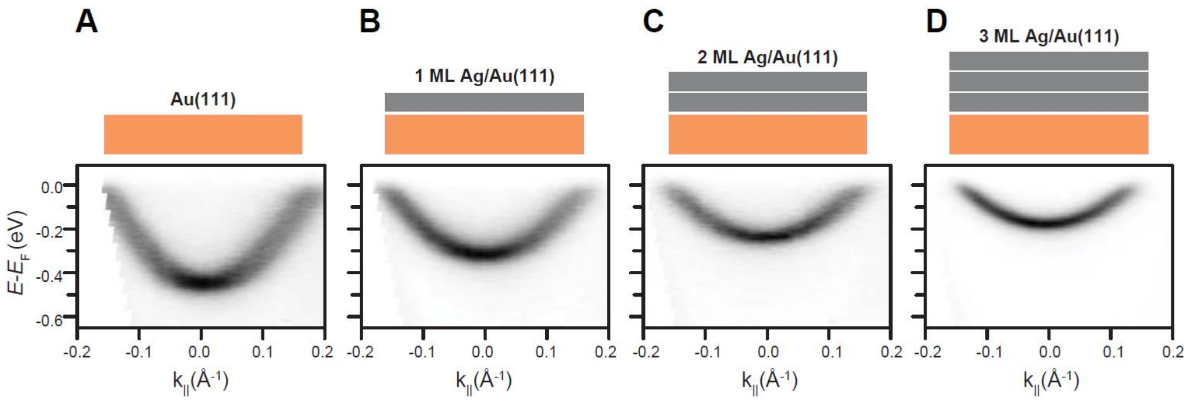

Supplementary Figure 7│Modification of the Au(111) 2DEG state with Ag film thickness. ARPES maps along $\overline{\Gamma M}$ measured directly on Au(111) in (A), after 1 ML Ag deposition in (B), 2 ML in (C), and 3 ML in (D) measured at 130 K. The surface state shifts its energy towards the Fermi energy in defined steps and increases its effective mass. For fractional coverages of a multiple of a ML, we observe a broadened state that is partly composed of contributions from the adjacent lower and upper layers. Thus, it is rather easy to discriminate the optimal thicknesses when using gradient coverage preparation conditions. The measured results are in perfect agreement with Ref. 4.

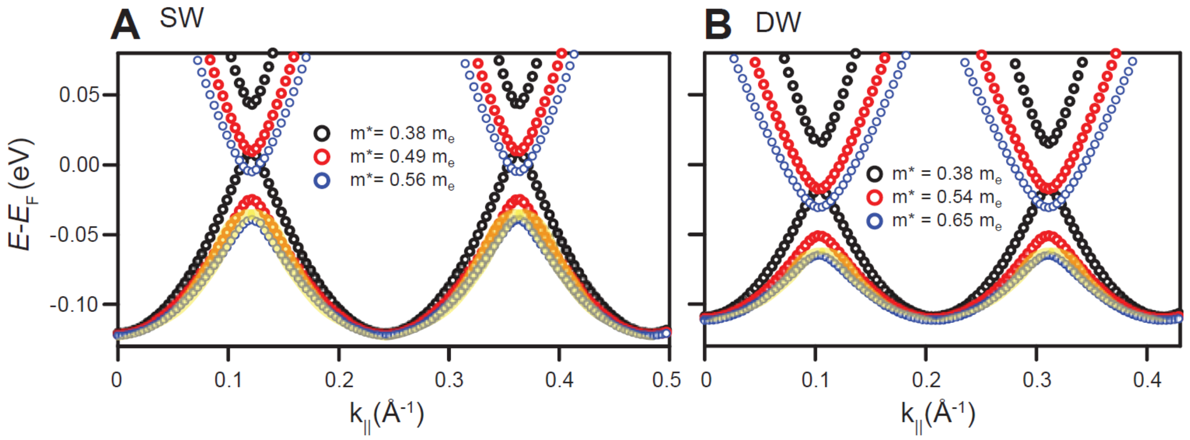

Supplementary Figure 8│Influence of the effective mass *m upon the calculated band structure for the same geometry and molecular potential V_{eff} using EBEM**. (A) Calculated electronic bands for the SW geometry using the effective masses of 0.38 m_e [pristine 3 ML Ag/Au(111) case, black], 0.49 m_e (optimal agreement for both ARPES and STS, red), and 0.56 m_e (best fit for only the ARPES data but deviating slightly from the STS, blue). (B) Calculated electronic bands for the DW geometry using the effective masses of 0.38 m_e [pristine 3 ML Ag/Au(111) case, black], 0.54 m_e (optimal agreement for both ARPES and STS, red), and 0.65 m_e (best fit for only the ARPES data but deviating slightly from the STS, blue). The results of these calculations can be compared with the experimental band structure represented as yellow transparent traces. It is observed that the pristine $m^* = 0.38\ m_e$ deviates substantially from the experimental bands and larger effective masses are required to fit all the experimental data. The differences found between the red and blue calculations in (A) and (B) falls practically within the experimental error.

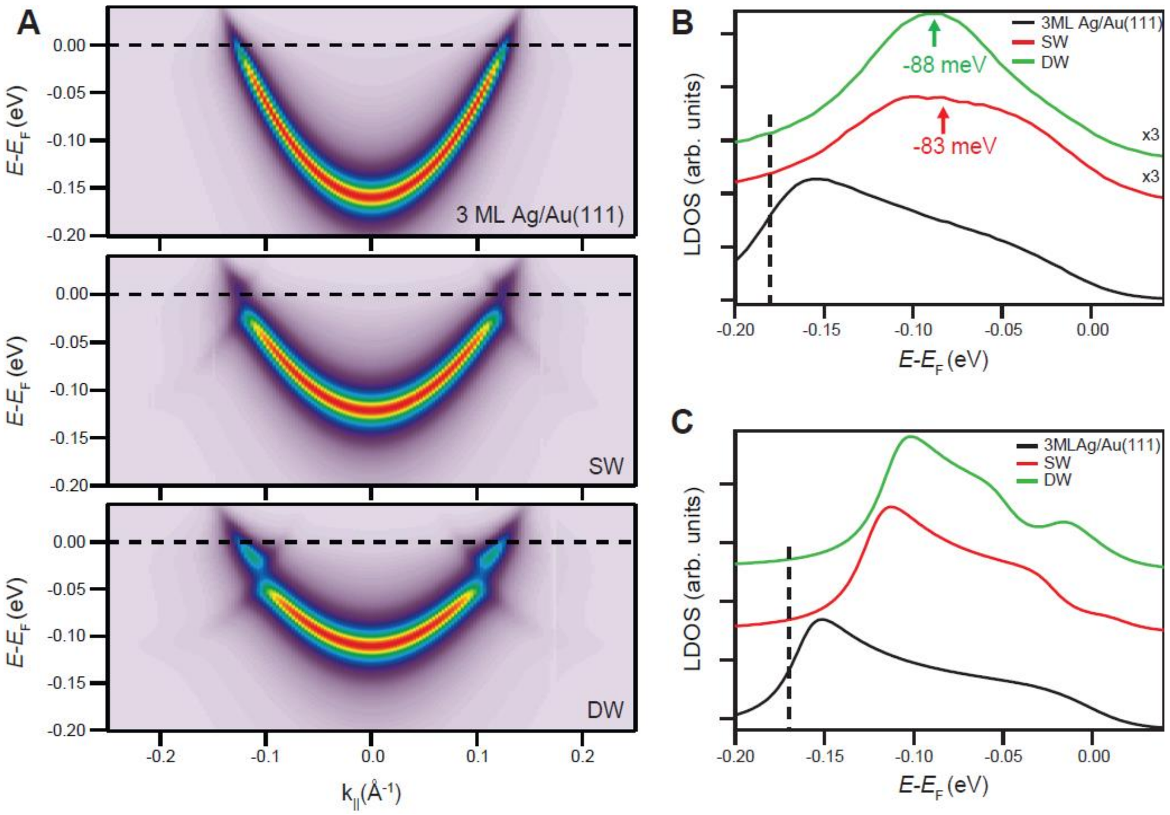

Supplementary Figure 9│Calculated EBEM band structures and its corresponding angle integration and comparison to the ARPES data. (A) EBEM simulated ARPES energy dispersion curves (EDCs) along $\overline{\Gamma M}$ high symmetry direction for the pristine 3 MLAg/Au(111), SW and DW cases. Such bands agree exquisitely with experimental ARPES data shown in Fig. 3 of the main text and show weak side features away from the prominent spectral function close to $k_{||} = 0$. However, in the simulations the gap between n = 1 and n = 2 is better defined than in the experiments. The possible reason is the ubiquitous inhomogeneities present when these networks are formed that broadens our experimental spectra, increases the background and washes out the gaps. The experimental angle integrated ARPES spectra from the raw data of Fig. 3 of the main text shown in (B) can be directly compared to the corresponding angle integration case shown in (C). The agreement for both experimental and theoretical integrations is satisfactory and the peaks extracted in (B) for SW (-83 meV) and DW (-88 meV) are indicative of their bandwidth difference. The line shapes are similar to (C), but the overall experimental shapes of the SW and DW have a larger background contribution and are more featureless.

Supplementary References

[1] T. T. T. Bui, S. Dahaoui, C. Lecomte, G. R. Desiraju, and E. Espinosa, The Nature of Halogen···Halogen Interactions: A Model Derived from Experimental Charge-Density Analysis , Angew. Chemie Int. Ed., **48**, 3838-3841, (2009).

[2] S. Kawai, A. Sadeghi, F. Xu, L. Peng, A. Orita, J. Otera, S. Goedecker, and E. Meyer, Extended Halogen Bonding between Fully Fluorinated Aromatic Molecules, ACS Nano, **9**, 2574-2583, (2015).

[3] T. Clark, M. Hennemann, J. S. Murray, and P. Politzer, Halogen bonding: the σ-hole, J. Mol. Model., **13**, 291-296, (2007).

[4] H. Cercellier, C. Didiot, Y. Fagot-Revurat, B. Kierren, L. Moreau, D. Malterre, and F. Reinert, Interplay between structural, chemical, and spectroscopic properties of Ag/Au(111) epitaxial ultrathin films: A way to tune the Rashba coupling, Phys. Rev. B, **73**, 195413, (2006).
